# Relationship between Oxidative Stress and Endometritis: Exploiting Knowledge Gained in Mares and Cows

**DOI:** 10.3390/ani12182403

**Published:** 2022-09-13

**Authors:** Raffaele Boni, Stefano Cecchini Gualandi

**Affiliations:** Department of Sciences, University of Basilicata, Campus Macchia Romana, 85100 Potenza, Italy

**Keywords:** mare, cows, endometritis, oxidative stress, ROS detection

## Abstract

**Simple Summary:**

Endometritis is a widespread pathology in mares and cows and one of the leading causes of reproductive efficiency decline. Although localized in the innermost layer of the uterus, this inflammation involves the release of bioactive molecules and by-products related to molecular damage. Among the bioactive substances, a primary role is played by the reactive oxygen and nitrogen species, which, while exerting an antimicrobial effect, can amplify the inflammation, sometimes causing unwanted and self-aggravating effects. Part of these substances enters the bloodstream, with implications that range from sustaining distant effects on organs and tissues to being used as diagnostic biomarkers. The research carried out on this matter in cows and mares has maintained certain independence due to its species specificity. This review aims to collect and analyze the information available in these species to enhance diagnostics and provide new insights to prevent and treat this pathology.

**Abstract:**

The etiopathogenesis of endometritis in mares and cows differs significantly; this could depend on a different sensitivity and reactivity of the uterus but also on endocrine and rearing factors and different stress sources. In both species, microorganisms and the immune system play a primary role in the generation of this pathology. Microbiological and cytological tests support clinical examination and significantly improve diagnostic accuracy. For both species, during the inflammation, immune cells invade the endometrium and release bioactive substances to contrast primary or secondary pathogen contamination. These molecules are traceable to cytokines, chemokines, and prostaglandins as well as reactive oxygen and nitrogen species (ROS and RNS), collectively known as RONS. The RONS-mediated oxidation causes morphological and functional alterations of macromolecules, such as proteins, lipids, and nucleic acids, with the consequent production of derivative compounds capable of playing harmful effects. These bioactive molecules and by-products, which have recently become increasingly popular as diagnostic biomarkers, enter the bloodstream, influencing the functionality of organs and tissues. This review has collected and compared information obtained in cows and mares related to the diagnostic potential of these biomarkers that are assessed by using different methods in samples from either blood plasma or uterine fluid.

## 1. Introduction

Chronic endometritis is a widespread and well-studied disease in livestock, such as cattle and horses, in which it causes significant economic losses due to a dramatic decline of fertility [1,2]. In both species, many studies have been undertaken to clarify the pathogenetic mechanisms of this disease and much information has been obtained. We believed that, from the analysis of this information, useful insights could be gained to prevent and treat this pathology. An experimental comparison between mares and cows on the pathogenesis of endometritis has been previously carried out using both in vivo and in vitro approaches [3]. The results highlighted common pathogenetic mechanisms but a different reactivity to the induced stimuli between species. In particular, following an interleukin (IL)-8 stimulation, the ability to generate reactive oxygen species (ROS) was significantly upregulated in vivo in both bovine and equine uterine neutrophils (PMN). However, after in vitro PMN migration, this ability was upregulated in horse and downregulated in cattle. In some cases, this pathology is clinically evident; in other cases, it is asymptomatic, making identification and resolution more difficult.

The diagnosis of chronic endometritis in both species involves clinical, cytological, and microbiological evaluations. A different approach could derive from the finding of inflammatory products detectable in the uterine fluid, as well as in the blood serum. Oxidative-derived products and substances contributing to the redox potential could represent significant biomarkers and ensure control of the inflammatory state of the endometrium. By analyzing the induced changes and the inflammatory products of endometritis detectable in the uterine fluid or in the peripheral blood, it will be possible to obtain useful diagnostic information that emerged from the studies carried out on these two species.

## 2. The Oxidative Profile and Its Alteration (Oxidative Stress) in Biological Systems

Molecular oxygen (O_2_) is vital for aerobic organisms; however, the by-products resulting from its consumption can cause oxidative damage to macromolecules and play a role as disease-causing agents. This occurrence is known as the “oxygen paradox” [4]. The oxygen by-products, i.e., reactive oxygen and nitrogen species (ROS and RNS) and collectively indicated as RONS, are endogenous, highly reactive, oxygen- and nitrogen-carrying molecules. They are mainly produced as a consequence of oxidative processes at the mitochondrial respiratory chain [5,6]. In addition to mitochondrial supply, RONS are produced by several enzymes such as NADPH oxidases (NOXs), cytochrome P450, xanthine oxidase, nitric oxide synthase (NOS), and at the peroxisomal level [7]. Among ROS, superoxide anion (O_2_^•−^) and hydrogen peroxide (H_2_O_2_) are the first oxidants to be produced by mitochondria. However, many other oxidants derived from cellular metabolism have been described. In particular, ROS includes other oxygen radicals, such as hydroxyl (OH^•^), peroxyl (RO^•^) and hydroperoxyl (HO_2_^•^), and other non-radical oxidizing molecules, such as hypochlorous acid (HOCl), hypobromous (HOBr), hypoiodous acids (HOI), and ozone (O_3_), that can be easily converted into radicals. Among RNS-bearing oxygen, nitric oxide radical (NO^•^), nitrogen dioxide radical (NO_2_^•^), nitrite (NO_2_^−^), and peroxynitrite (ONOO^−^) are worthy of mention [6,8]. RNS derive from nitric oxide (NO), which is generated during the breakdown of arginine into citrulline by NADPH-dependent enzymes [9]. Mitochondria, as well as other cellular components, are equipped with enzymes capable of maintaining oxidants below harmful levels [10]. 

Small quantities of RONS are essential for the correct performance of physiological activities, such as redox signaling in mitochondria. However, excessive RONS production, related to the cellular response to physical, chemical, and biotic stress caused by environmental conditions, xenobiotics, pollutants, cytokines, and pathogen invasion [11,12], can be detrimental to the welfare or even put at risk the survival of the organism. This threat occurs when the quantities of oxidant exceed the buffering capacity to counteract the deleterious oxidant effects, with the primary risk of losing the homeostatic ability to maintain or recover the redox balance, i.e., the correct ratio between oxidant and antioxidant substances. It is only after the breakdown of the redox equilibrium that the organisms can experience oxidative stress (OS), with the prevalence of oxidant radicals causing dangerous oxidative changes of macromolecules.

OS is generally defined as an imbalance between the amount of RONS produced during the cellular metabolic processes and the cellular capability to detoxify reactive intermediates or to repair the consequent damage by an appropriate antioxidant defense system (ADS). In recent years, the improved knowledge of the role of oxidants in physiological processes has suggested the more complex concept of oxidative “eustress/distress” [13]. Thus, the oxidative challenge, which is involved in adaptive or regulatory signaling, is placed in oxidative eustress (OeS), while the occurrence of high RONS levels, oxidative damage, and apoptosis induction is placed in oxidative distress (OdS) [13]. The ADS is essential in protecting against the oxidation of cellular targets such as DNA, lipids, and proteins, with damages associated with either structural or functional properties. The ADS includes a combination of endogenous enzymatic, as superoxide dismutase (SOD), catalase (CAT), glutathione peroxidase (GPx), thioredoxin (Trx), and non-enzymatic, as thiol groups, uric acid, bilirubin, substances and exogenous substances, as selenium, zinc, vitamin C and E, carotenoids, and polyphenols, which act together to prevent or curtail the oxidative damage of macromolecules [5,14].

In proteins, the oxidative damage is mainly reflected in the irreversible modification of the protein backbone, chain fragmentation, and oxidation of the side chains of amino acids. These modifications are frequently responsible for the production of carbonyl moieties, mainly in the case of proline, arginine, and lysine side-chains [15]. The enzymes involved in oxidative processes lose their catalytic activity and this, in turn, leads to dysregulation of metabolic pathways. Oxidative lipid damage (lipoperoxidation, LPO) is mainly directed against unsaturated fatty acids; in fact, double bonds in fatty acids represent intrinsic regions with high oxidant reactivity. Among unsaturated acids, polyunsaturated fatty acids (PUFA), such as membrane phospholipids, represent the main target because their double bonds are frequently in conjugated geometry [15]. Moreover, the lipid damage leads to the release of further radicals (alkyl-) or aldehydes, which in turn may be responsible for further cellular threats. LPO causes alterations of the membrane structure, damaging its integrity and affecting its fluidity [16]. Among aldehydes, malondialdehyde (MDA) and 4-hydroxynonenal (4-HNE) represent the most used biomarkers for assessing the degree of lipid peroxidation in OdS. These aldehydes can form adducts with the proteic-NH_2_ groups and with the DNA bases, in turn, capable of determining mutations [16]. Oxidative DNA damage may induce multiple alterations, such as base and sugar lesions, single- and double-strand breaks, abasic sites, and DNA-protein crosslinks, due to the oxidative reactions with nitrogenous bases and deoxyribose. Oxidative DNA damage, usually associated with aging, causes mutations, cellular apoptosis and necrosis, and is involved, together with the oxidative damage of lipids and proteins, in the pathogenesis of human degenerative lifestyle-related diseases, such as Alzheimer’s disease and Parkinson’s disease, amyotrophic lateral sclerosis, some hereditary diseases, hypertension, diabetes mellitus, ischemic diseases, and cancer [12,16,17].

### 2.1. OS Assessment

Several analytical methods are available for evaluating OS in biological samples. These tests allow monitoring of OS biomarkers but the lack of species-specific reference values in veterinary medicine does not always allow the identification of the OS condition [18]. This review will consider only the tests that are primarily intended for use in clinical studies mainly based on spectrophotometric or immune-enzymatic assays that may be performed by clinical auto-analyzers allowing rapid and inexpensive data collection. Moreover, these tests are often available as commercial kits, where execution is facilitated by ready-to-use reagents.

#### 2.1.1. Redox Potential

Owing to their high instability, RONS are indirectly assessed by the more stable and longer half-life ROS and RNS derivatives. Among them, H_2_O_2_ and lipid hydroperoxides (LOOHs) can be used for ROS detection, whereas nitric oxide final metabolites (NO_x_), i.e., the sum of nitrite (NO_2_^−^) and nitrate (NO_3_^−^), and peroxynitrite (ONOO^−^), can be used for RNS detection in biological samples. Serum H_2_O_2_ levels can be directly measured by using molybdate that, reacting with ROS, forms a peroxomolybdic acid complex read with a spectrophotometer [19]. LOOH, i.e., the primary oxidation products of PUFAs, can be mainly detected by using two different methods, known as the reactive oxygen metabolites (ROMs) and the total oxidant status (TOS) assays [20,21]. These methods are based on the oxidation of N,N-diethyl-para-phenylenediamine (DEPPD) and Fe^2+^–*o*-dianisidine complex in ROMs and TOS assays, respectively. Both are widely used on blood serum and plasma [18,22,23,24] as well as on saliva, exhaled breath condensate, and seminal plasma [25,26,27]. Although ROMs and TOS assess the same molecular targets, they report two independent and unrelated measures [18,21,28]. This independency is further corroborated by the analytical interference between ROMs and ceruloplasmin (CP) oxidase activity [21,29]. As additional test in total oxidant assessment, the activity of myeloperoxidase (MPO), can be considered a valid oxidant biomarker. MPO is a peroxidase enzyme that is abundantly expressed in PMN and uses H_2_O_2_ to catalyze the production of hypohalous acids playing an intense antimicrobial activity during inflammation. This enzymatic activity can be evaluated on MPO-H_2_O_2_ oxidation of 3,3′,5,5′-tetramethylbenzidine hydrochloride (TMB) and is well correlated with TOS [18]. NO_x_ is quantified in deproteinized samples by using the reaction between nitrite, sulfanilamide (SULFA), and N-naphthyl-ethylene-diamine (NEDA) that forms an AZO product, following the reduction of nitrate to nitrite by vanadium(III) chloride (VCl_3_) [30]. ONOO^−^, a reactive biological non-radical oxidant, derives from free radical precursors, the superoxide (O_2_^•–^) and the nitric oxide (NO^•^), and it is responsible for protein, DNA, and lipids nitration. Despite having a longer half-life than its precursors, ONOO^-^ cannot be considered a stable molecule, unlike NO_x_. It can be detected by several methods; one of them is based on the ability of ONOO^−^ to nitrate the phenol, resulting in the formation of a nitrophenol complex [31].

The ADS is equipped with various endogenous or exogenous molecules, with either enzymatic or non-enzymatic activity. So far, the antioxidant status was evaluated on the activities of each antioxidant substance. Actually, the whole antioxidant evaluation represents an assessment of the overall antioxidant status summing up interactions between the different antioxidant molecules. Usually, the antioxidant potential of a biological sample is measured as the content of free radicals scavenged by a test solution [32]. This represents the integrated cumulative actions of all the antioxidant substances of the biological sample. The ferric reducing ability of plasma (FRAP) assay [33] is based on the principle of the reduction of the ferric-tripyridyltriazine complex to the ferrous form. This method is sensitive to uric acid, representing approximately 60% of the antioxidant capacity in human samples [34], as well as to low molecular weight antioxidants, such as α-tocopherol, bilirubin, and ascorbic acid; however, it does not consider the SH group-containing antioxidants, such as glutathione (GSH) and albumin. The improved ABTS radical cation (ABTS^•+^) assay [35], also known as trolox equivalent antioxidant capacity (TEAC), is based on the shift of the 2,2′-azinobis(3-ethylbenzothiazoline- 6-sulfonic acid) radical cation to its reduced form in relation to the antioxidant content of the sample. This method is affected by the uric acid, even if less than FRAP assay, and detects albumin, carotenoids, α-tocopherol, ascorbic acid, glutathione, and phenolic compounds. Free radical scavenging activity (FRSA) uses free radical traps to assess the scavenging capacity of a sample and is based on the reduction of the DPPH^•^ to 1,1-diphenyl-2-picryl hydrazine [36]. The oxygen radical absorbance capacity (ORAC) uses fluorescein as substrate and 2,2′-azobis(2-amidinopropane) dihydrochloride (AAPH) as oxidant generator; the decrease in fluorescence intensity is related to the antioxidant capacity of the sample [37]. Unlike the aforementioned methods that are read with a spectrophotometer, this test is read with a spectrofluorometer. According to Cao et al. [38], the main molecules involved in ORAC detection are α-tocopherol, ascorbic acid, uric acid, bilirubin, and, to a lesser extent, albumin. Unfortunately, often the results obtained with these tests evaluating the total antioxidant activity are not comparable between each other [18,34]. However, in donkey seminal plasma, data from several antioxidant assays were well correlated with each other and some of them were candidates as reliable markers of sperm functionality [25]. The lack of correlation between these assays may depend on the analytical technologies used or the different reactivity between the mixture of antioxidant substances and the analytical solutions [28,34,39]. Therefore, the simultaneous evaluation of different analytical assays and their comparison play a pivotal role in detecting the true redox potential status in biological samples. Furthermore, the ratio between total oxidant and antioxidant levels provides the oxidative stress index (OSI), an indicator of the redox balance [40]. Finally, the evaluation of total thiol (sulfhydryl group, -SH) levels (TTLs) can add further information on the antioxidant potential of biological samples, since the thiol protein groups, at least in healthy humans, represent approximately 50% of the plasma total antioxidant barrier [41]. TTL assay is based on the interaction between thiols and 5,5′-dithiobis-(2-nitrobenzoic acid) (DTNB) to form a highly colored anion [42]. Since thiol groups form disulfide bonds upon oxidation, the improved method proposed by Erel and Neselioglu [43] represents a significant step forward in the study of dynamic thiol/disulfide balance. In this method, a native thiol is obtained by reducing disulfide bonds to free thiol groups by NaBH_4_, and total thiol concentrations are synchronously measured. Subsequently, the disulfide bond level can be indirectly calculated as the half value of the difference between the total thiol and the native thiol levels.

#### 2.1.2. Protein Oxidative Damage

Protein carbonylation refers to the oxidation of protein side chains that introduces carbonyl groups (CGs) in proteins and relies on different mechanisms. This mainly takes place by either direct ROS-mediated oxidation of the protein backbone, leading to the formation of protein fragments with an N-terminal α-ketoacyl amino acid residue, or by oxidation of the side chains of lysine, proline, arginine, and threonine residues into ketone and aldehyde derivatives. However, protein carbonylation may be also played by products of lipid peroxidation, such as 4-hydroxy-2-nonenal, the conjugation with reducing sugars (glycation), or their oxidation products (glycoxidation) [44]. Several methods have been proposed to evaluate the protein oxidative damage. Among them, the protein carbonyl (PC) content and the advanced oxidation protein products (AOPPs) are commonly applied tests. PC content is widely used for its simplicity and reliability [45] and is based on the reaction between 2,4-dinitrophenylhydrazine (DNPH) forming a Schiff base to produce dinitrophenylhydrazone products. In humans, ROS-mediated protein carbonylation is considered a good indicator of OS-related diseases, such as aging degenerative disorders and ischemic stroke [46,47]. In veterinary medicine, the PC assay has been associated with some pathologies [48], as well as used to evaluate the fitness status following regular exercise training in equine athletes [49]. AOPP assay evaluates the oxidative protein modifications by detecting dityrosine-containing protein crosslinked products, generated by the reaction of plasma proteins, mainly albumin, with chlorinated oxidants, such as hypochlorous acid and chloramines. Therefore, this is the reference method for assessing the protein oxidative damage due to Cl-based oxidizing agents. The original method, proposed by Witko-Sarsat et al. [50] and based on the spectrophotometric reading after acid treatment of the sample, has now been improved by Hanasand et al. [51] replacing the acetic acid reagent with a citric acid solution to avoid lipid precipitation. AOPP assay has been used in humans mainly to check protein damage in renal failures and endometrial diseases [50,52]. In veterinary medicine, AOPP level is considered an indicator of acute inflammation and OS, and it has been associated with embryonic losses in dairy cows [53], and with the content of both ROS- and RNS-derived substances in jumper horses [40]. Since AOPPs show a long half-life and activate the neutrophil and monocyte oxidative metabolism, they are involved in a prolongation of the OS and inflammatory response [54,55].

#### 2.1.3. Lipid Oxidative Damage

Lipid peroxidation (LPO) is largely used as a marker of ROS-mediated damage to cell membranes and is mainly detected by thiobarbituric acid reactive substances (TBARS) assay [56]. LPO can be expressed in terms of malondialdehyde (MDA) equivalents (μM) obtained from MDA generation with 1,1,3,3-tetraethoxypropane (TEP) hydrolysis [57]. This method, however, shows a low specificity and accuracy as well as artifact occurrence. In fact, aldehydes other than MDA, reacting with TBA, produce derivatives that absorb light in the same wavelength range as MDA [58]. Alternatively, MDA can be more accurately measured using either high-performance liquid chromatography [59] or gas chromatography–mass spectrometry [60]. TBARS values have been well associated with TOS and NO_x_ levels in athletic horses and, hence, are proposed as reliable indicators of OS [40]. Moreover, in the last few decades, new peroxidative-derived molecules were found. The oxidative attack of PUFA component of cell membranes generates a family of α,β-unsaturated reactive aldehydes, such as 4-hydroxy-2-nonenal (4-HNE) and prostaglandin-like end-products (isoprostanes, IsoPs). Among these products, 8-isoprostaglandin F2α (8-iso-PGF2α) and 4-HNE are considered accurate OS biomarkers. 4-HNE is the result of peroxidation of ω-6 PUFAs, essentially arachidonic (AA) and linoleic (LA) acid, the two most represented fatty acids in cellular membranes, while 8-iso-PGF2α is generated following the peroxidation of several PUFAs, including AA, eicosapentaenoic acid (EPA), adrenic acid (AdA), and docosahexaenoic acid (DHA) [61]. The use of PGF2α assessment is still scarce due to technical and economical limitations [62,63]. To overcome these hitches, immune-enzymatic methods for PGF2α assessment have been developed; these, however, often detect data strongly overestimated and poorly correlated with those obtained with the reference methods, involving gas chromatography–mass spectrometry (GC–MS) and tandem liquid chromatography-tandem mass spectrometry (LC/LC–MS/MS) [63]. Measured by immune-enzymatic methods, 4-HNE has been mainly used in human medicine [64], whereas in veterinary science, its interest is still limited to assessing toxic by-products released during meat storage [65]. During OS, 4-HNE undergoes several reactions to form adducts with target macromolecules (proteins, peptides, phospholipids and nucleic acids). These structural modifications are further responsible for functional alterations of the biological activity of the involved macromolecules with cytotoxic, mutagenic, genotoxic, and signal effects [66].

#### 2.1.4. DNA Oxidative Damage

Several methods are available to assess DNA damage due to oxidative processes. They are mainly based on cellular analyzes, and are widely used for sperm quality assessment as the acridine orange (AO) test, the sperm chromatin structure assay (SCSA), the aniline blue, the toluidine blue, and the chromomycin A3 (CMA3) staining, the in situ nick translation (ISNT), the sperm chromatin dispersion (SCD) test, the terminal deoxynucleotidyl transferase mediated deoxyuridine triphosphate nick end labelling (TUNEL) assay and the single-cell gel electrophoresis (comet) assay (for review, see [67]). Single- or double-stranded breaks and base oxidations, as the 8-oxoguanine-DNA-glycosylase (hOGG), represent direct evidence of DNA damage. Indirect evidence, however, can be assessed by evaluating the cellular DNA-repair potential [68,69]. The 8-hydroxy-2′-deoxyguanosine (8-OHdG) originating from the deoxyguanosine hydroxylation is a well-known biomarker of DNA oxidative damage [70]. Since the DNA repair system excises 8-OHdG residues to preserve DNA integrity, 8-OHdG levels can be found in biological fluids, such as blood and urine samples [71]. Previously, 8-OHdG levels were measured by HPLC with electrochemical detection (HPLC-EC) [70]; actually, ELISA kits detecting 8-OHdG are available allowing the use of this biomarker for investigating the OS involvement in many human diseases [72]. Additionally, in veterinary medicine, 8-OHdG is more and more assessed for evaluating OS-mediated DNA damage in either biotic or abiotic diseases [73,74,75].

## 3. The Role of OS in Inflammatory Processes

Inflammation is the physiological adaptive response of the organism against injuries of different origin aimed to restore or maintain homeostasis through the induction of various repair mechanisms. It involves various types of cells, including polymorphonuclear and mononuclear cells and fibroblasts. The correct regulation of repairing mechanisms is fundamental in the prevention of undue amplification of the initial inflammation, which may lead to further tissue lesions and disease development. The relationships between OS and inflammation pathways are based on finely regulated mechanisms [76]. As neutrophils reach the inflammatory site, the superoxide anion radicals (O_2_^•−^) activate the “respiratory burst”, an event generated through the univalent reduction of molecular oxygen by the NADPH oxidase enzyme complex. Following the spontaneous conversion of O_2_^•−^ to H_2_O_2_, chlorine- and bromine-derived oxidants are generated by MPO catalytic activity [77]. During the respiratory burst, immune cells also generate RNS via iNOS and proinflammatory cytokines, such as tumor necrosis factor (TNF)-α, IL-1β, and IL-6. This occurrence is amplified by proinflammatory cytokines released from damaged tissues, which in turn cause the activation of ROS- and RNS-generating enzymes [78,79]. All inflammatory adaptive events aim to confine the inflammation and repair the damaged tissue with homeostasis restoring [80]. If these conditions are not reached, the inflammation becomes chronic [81]. This alters the redox balance by increasing the levels of oxidants and lowering of the buffering capacity [82] and, therefore, allows further macromolecule damages. In addition, several molecules resulting from oxidative damage, such as isoprostanes, aldehydes, modified proteins, and MDA-adducts, activates inflammatory mediators fueling inflammation in a self-perpetuating cycle [83]. The simultaneous presence of chronic inflammation and OS in many chronic and degenerative human diseases demonstrates the close association between these events [12,16,17]. However, it is difficult to clarify if OS is the cause or the consequence of the pathogenetic machinery.

## 4. Endometritis

In cattle, endometritis is usually a calving-associated disease that remains confined within the uterus without systematic involvement of the health. Hence, it is clearly distinguished from metritis, an inflammation that involves all components of the uterus with signs of systemic illness such as fever, decreased milk yield, dullness, and toxemia. These two uterine diseases can also be classified in different timescales: metritis occurs within 21 days after parturition, and endometritis later on [2]. Endometritis can be clinical (CE) and subclinical (SCE). The former is characterized by the presence of mucus-purulent uterine discharge, the latter does not manifest evident symptoms and can be diagnosed mainly by cytological and microbiological findings [2]. In mares, even if postpartum endometritis is frequent, the main event triggering endometritis is insemination, either natural or artificial (post-breeding endometritis, PBE) [84]. Reproductive anatomy, defective myometrial contractility, lowered immune defenses, overproduction of mucus, inadequate lymphatic drainage, or a combination of these factors will predispose the mare to PBE [85]. It can be classified as acute and chronic as well as clinical (CE) and subclinical (SCE) [86].

Regardless of the predisposing conditions that deal with an individual susceptibility [87], endometritis etiology is mainly due to bacterial or fungal components [88,89] either as a primary or secondary occurrence. In the former, specific, and often, sexually transmitted microorganisms are responsible for the disease; in the second, germs widely spread outside can grow within a uterine environment unable to counteract their development. The origin of this contamination is complex and can derive from poor hygiene at the delivery, from incomplete expulsion of the placenta, from pneumo- or uro-vaginal conditions resulting from a particular conformation of the perineum, characteristics of the individual or even consequent to a reduction in fat accumulation in the pelvis cavity [86]. However, the presence of a number of bacteria that are normally resident in the uterus in dormant form and able to activate in the presence of predisposing conditions has also been demonstrated [90]. The sterility of the uterus after a certain period of delivery seems to be denied by recent findings demonstrating the residence of bacteria in the uterus even during pregnancy [91,92]. Additionally, the semen is very rarely bacterial-free and, therefore, the introduction of bacteria at the time of insemination, whether natural or artificial, represents a concrete pathogenetic hypothesis [86].

Although endometritis can resolve spontaneously, numerous therapies have been put in place for its treatment, with results that are not always favorable. In some cases, this disease becomes chronic and resistant even to targeted antibiogram-based therapies. To treat these animals, alternative therapies have been developed ranging from the use of hormones, to substances stimulating the immune defenses to topical disinfectants or even fizzy drinks [93]. Antiseptic solutions, in general, evoke an acute inflammation of the endometrium, being able to involve necrosis in the most superficial layer of the uterine mucosa [94] together with the microbicidal activity that is often responsible for this inflammatory condition. The endometrial and inflammatory cells in response to this strong insult enter a state of stress and release inflammation products such as cytokines, ROS, and prostaglandin F2α [95]. If the treatment-induced damages are not excessive and the concrete possibility of endometrial fibrosis does not occur [96], the endometrium undergoes regeneration starting from surviving stem cells present in the basal layer of the epithelium [97]. Often, however, the microorganisms involved in this pathology show forms of resistance to the treatments used [98]. An innovative approach aimed at counteracting the antibiotic-resistance of the bacteria responsible for endometritis proposes the use of silver nanoparticles [99]. This treatment involves the release of ROS as a primary bactericidal mechanism but plays inevitable side effects on the endometrium welfare due to its high toxicity.

The diagnosis of endometritis can be made by clinical, microbiological and cytological examination. Clinical examination is based on the ability of the veterinary practitioners to appreciate the presence of fluid in the uterus at either transrectal palpation or ultrasound examination as well as the presence of mucus discharge from the vulva or the cervix at vaginoscopy. The microbiological analysis is based on the collection, under sterile conditions, of material from the uterus that is, subsequently, sown in microbiological plates to allow the selective growth of the germs present. Intrauterine sampling is generally obtained by a double-guarded swab or uterine flushing fluid. A high correlation was found between positivity to the microbiological test, the finding of endometritis and fertility [100]. The cytological examination is based on the collection, staining and microscopic observation [101,102] or fluorescence spectroscopy/microscopy [103] of uterine cells to identify the presence of immune cells; these are mainly represented by PMN both in mares and cows, whereas by plasma cells in humans [104]. This would suggest that the primary immune response in cattle and mare is cell-mediated while it is humoral in humans. The finding of a variable amount of PMN mixed in the uterine cells ranging from 0.5–1% in the mare to 5–18% in the cow ensures the diagnosis of endometritis. The large variability described in the cow is essentially attributable to the fact that the PMN as well as the bacterial content in the uterus decreases as the distance from calving increases. In fact, the PMN cut-off value for endometritis diagnosis decreases from >18% on 21–33 d post-partum (pp) to >10% at 34–47 d pp and >5% later [105,106,107]. The cell sampling can be carried out by double-guarded swab, cytobrush, cytotape and uterine flushing. Each of these options has pros and cons: (i) the swab has a limited ability to recover cells [108]; (ii) the cytobrush and the cytotape provide endometrial samples with similar diagnostic values involving a limited area of the endometrium, the second with less blood contamination than the first [109]; and (iii) uterine flushing mainly recovers exfoliated mucosal cells, with a high prevalence of debris [108].

## 5. The Role of the Immune System in the Pathogenesis of the Endometritis

In addition to infections, the immune system plays a pivotal role in this pathology in both species. By intervening in defense of the endometrium, the immune cells release pro-inflammatory cytokines and chemokines, which can themselves become a part of the inflammatory process, creating self-aggravating conditions [110]. It is currently unknown why endometritis affects some individuals rather than others. Individual reactivity was tested in mares by experimental infection with *S. zooepidemicus*, distinguishing between mares susceptible and resistant to endometritis [87]. Exploiting individual receptivity to endometritis, Canisso and colleagues found that mares susceptible to PBE, or those with difficulty clearing infection/inflammation, have a deficient immune response and compromised physical mechanisms of defense against infection [111].

In general, an endometritis-associated variation in blood leukocyte content during endometritis is controversial in either cows or mares [112,113,114]. In particular, among the authors who recorded differences, in cows with SCE, Duvel and colleagues [112] found a significantly higher number of blood mononuclear cells and PMN. Among mononuclear cells, the number of B cells, NK cells, and CD172a-positive monocytes was significantly elevated. PMN are the earliest and most relevant phagocytic cell type recruited from the peripheral circulation in response to pathogen challenges. Following pathogen recognition, immune cells release pro-inflammatory molecules including TNF-α, interleukins (IL-1, IL-6, IL-8, IL-12), and NO [115]. These molecules stimulate the recruitment and activation of more immune cells and the hepatic secretion of acute-phase proteins [116]. NO has been detected in the mammalian uterus with endometritis causing uterine relaxation [117]; it is influenced by steroid hormones [118] but may also change either the structure or the function of these hormones [119]. In mares, uterine accumulation of NO was found in PBE [120,121]. In addition, resistant mares had a relatively steady amount of total intrauterine NO over 24 h after breeding, while susceptible mares had a significant increase in total NO than resistant mares at either 6 and 12 h after breeding [121].

The change in leukocyte activities involves the phagocytic activity and the respiratory burst of both blood and uterine PMN. Mateus and colleagues [114], by studying cows in the peripartum period, found differences in the dynamics of activation of PMN which occurs almost simultaneously at the onset of mild and intense endometritis; however, the latter maintains this steady-state activation for longer. Additionally, evaluating the ability of PMN to react and engulf microorganisms, these authors hypothesized that a decrease in blood PMN respiratory burst activity in the early postpartum could be associated with increased susceptibility to early postpartum endometritis [114]. The reactivity of leukocytes during endometritis was also tested to evaluate the effectiveness of treatments with targeted action towards bacteria responsible for endometritis (cephalosporins as cephapirin) or enhancing the immune system (methisoprinol) [122]. As expected, the uterine infusion of methisoprinol increased the phagocytic and killing activity of phagocytes in the uterus. Conversely, the uterine infusion of cephapirin caused a reduction in the phagocytic and killing activity of phagocytes. A decrease in phagocytosis was also found when both treatments were simultaneously combined.

A possible association between the onset of this pathology, and the immune and nervous systems has also been taken into account [123]. Several studies provide evidence about the relevant role of the vagus nerve in regulating the innate immune function through the cholinergic anti-inflammatory pathway in response to bacterial infections [124]. The cholinergic anti-inflammatory pathway is mediated by acetylcholine that acts by inhibiting the production of TNF, IL-1, and macrophage migration inhibitory factor [125,126]. Comparing healthy cows and cows with CE and SCE, Siquera and colleagues [123] found that the activity of acetylcholinesterase in blood and lymphocytes increased in both CE and SCE groups; in addition, serum concentration of IL-1β was significantly higher in cows with endometritis.

## 6. Exploiting Oxidative and Metabolic Stress and Inflammatory Markers in Blood Serum and Uterine Fluid for Detecting Endometritis

**Figure 1:**
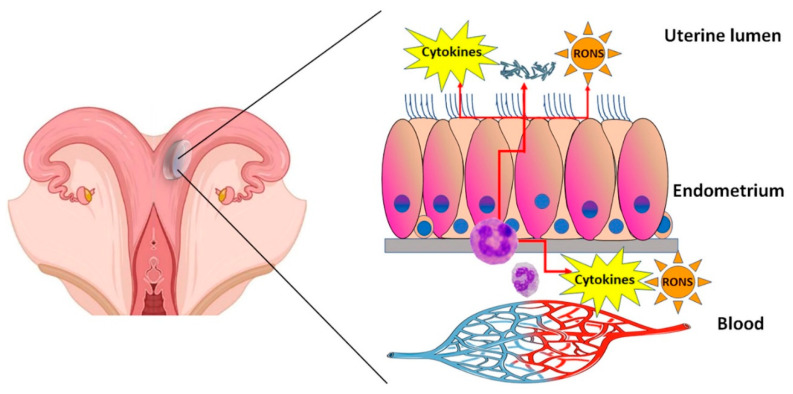
Schematic representation of the pathogenesis of endometritis in cows and mares. Immune cells, as polymorphonuclear granulocytes (PMN), moving from the bloodstream reach to the uterine mucosa following a chemotaxis attraction by microbial compounds, pro-inflammatory molecules and inflammation by-products. In endometrium, PMN carry out phagocytic and antimicrobial activity with the production of cytokines and reactive oxygen and nitrogen species (RONS), which pour into the uterine lumen and partly reach the bloodstream.

Although endometritis is defined as a disease confined within the uterine cavity, the products of inflammation are released from the uterine mucosa and accumulated in the uterine fluid. These substances, especially small molecules, such as RONS, could enter the bloodstream and spread in the body thus playing an unknown role in some diseases as well as acquiring a diagnostic value (Figure 1). In support of previously denied systemic effects, endometritis has been recently associated with a reduction in oocyte developmental competence. In fact, through experimentally induced endometritis with pathogenic bacteria, it was shown that oocytes collected by ovum pick-up had a lower ability to develop morulae after IVF in cows with endometritis than in control cows [127]. By in vitro stimulating PMN and monocytes of healthy cows with the plasma of cows with and without SCE, Duvel and colleagues [112] did not find differences in gene expression. However, the plasma from cows with SCE showed a significant increase in expressed messenger RNA copy numbers of CXCL8, CXCL1, and IL1B in intermediate monocytes. Analyzing the blood serum and uterine flushing fluids in cows during the first 40 d pp, Brodzki and colleagues [128] found that proinflammatory cytokines, such as TNF-a, IL-6, IL-10, and acute-phase proteins, such as haptoglobin (Hp) and serum amyloid A (SAA), showed time-dependent changes in serum, but primarily in uterine fluid, which may be used as reliable diagnostic indicators for SCE. Additionally, they suggest that the higher levels of IL-10 in cows with SCE may contribute to the weakening of local resistance mechanisms of the uterus and lead to the persistence of the inflammation in the postpartum period. Another study in cows [129], focusing on the evaluation of acute-phase proteins in the blood in relation to endometritis during 28–32 d pp, showed a significant increase in Hp, SAA, and ceruloplasmin in the presence of endometritis with levels proportionate to the severity of the disease. A significant correlation was also found between ceruloplasmin vs. Hp and SAA levels. In mares, the trend in acute-phase proteins may not reflect that observed in the cow. Following insemination of mares with frozen–thawed semen, the plasma concentrations of SAA, Hp, and fibrinogen did not increase above clinical threshold concentration and there were no differences between susceptible and healthy mares [130]. However, in a previous research [131] carried out in mares submitted to a controlled infection with an intrauterine infusion of *E. coli*, a significant increase in the acute-phase proteins, SAA and fibrinogen was found. Moreover, quantitative gene expression of SAA was significantly upregulated at 3 and 12 h post-insemination, and a significantly upregulated expression of IL-1β, TNFα, IL-8 and IL-10 was observed at 3 h post-insemination. Furthermore, the plasma concentration of SAA was significantly correlated to endometrial SAA expression.

### 6.1. Proteomics and Transcriptomics Analysis for Detecting Endometritis

A blood serum proteomic analysis of cows with and without cytological endometritis showed a differential abundance in 25 proteins out of 181 non-redundant proteins evaluated [132]; these included 4 binding protein-α and mannose-binding lectin 2 involved in immune responses. RNA extracted from endometrial biopsies at 7 and 21 d pp showed significant induction in inflammatory gene expression in all tested cows at 7 d pp [133]. However, 73 genes and 31 miRNAs were differentially expressed between healthy cows and cows that subsequently developed CE. While significant differential expression of 4197 genes in the transcriptome of healthy cows between 7 and 21 d pp showed the transition from a pro-inflammatory to tissue proliferation and repair status, only 31 genes were differentially expressed in cows with CE. This suggests that the inflammatory cascade activated in early postpartum is resolved in healthy cows within 21 d pp but this transition fails to occur in cows that develop CE. Furthermore, inflammatory activity is not confined to the uterus. Decreased circulating granulocytes and increased acute-phase protein expression levels were found, on 7 d pp, in the plasma of cows that developed CE. A subsequent study considered the transcriptomic activity of peripheral blood leucocytes and cells obtained from uterine biopsies of cows with and without endometritis at 45–55 d pp [134]. Nineteen differentially expressed genes associated with SCE were common to both circulating leucocytes and endometrial tissues. Among these genes, transcript abundance of immune factors *C3*, *C2*, *LTF*, *PF4*, and *TRAPPC13* were upregulated in SCE cows. Moreover, mRNA expression of *C3*, *CXCL8*, *LTF*, *TLR2*, and *TRAPPC13* was temporally regulated after calving in circulating leucocytes of healthy cows compared to cows with SCE. This observation might indicate an advantageous modulation of the immune system in healthy animals.

### 6.2. Metabolic and Oxidative Stress

Based on the possible role played by rearing conditions and metabolic stress in the etiopathogenesis of bovine endometritis, studies have been undertaken to find the clues contained in the metabolic profile associated with the onset of this disease. Blood serum markers for systemic inflammation and liver, mineral, and energy status were analyzed in healthy, CE, and SCE dairy cows during the first 35 d pp [135]. In most of the analyzed days, Hp levels were higher in cows with SCE and CE than in healthy cows. Albumin levels were lower in cows with CE and SCE than in healthy cows at 14 and 35 d pp, respectively. The total calcium levels were lower one week before calving in cows with SCE and at 7 and 14 d pp in cows with CE than in healthy cows. At 14 d pp, serum non-esterified fatty acids (NEFA), β-hydroxy butyrate (BHB), and globulin levels were higher, and IGF-1 lower in cows with SCE than cows with CE or healthy cows. This pattern reflects negative energy balance in peripartum cows and is associated with uterine disease and infertility [136]. In particular, low serum concentrations of insulin-like growth factor-1 (IGF-1) are associated with a risk of approximately 4-fold higher of developing cytological endometritis [137]. A relationship between subclinical ketosis and endometritis has been found in dairy cows; these diseases were associated together with significant variations in MDA and TAC levels [138]. These findings were confirmed in a very recent study [139], demonstrating that serum levels of BHB, and clinical ketosis are positively associated with the number of PMN found at endometrial cytological evaluation. However, an inverse relationship has been found between the number of PMN and serum levels of urea and albumin. This supports the hypothesis that SCE is associated with unsatisfied postpartum metabolic demands and metabolic inflammation. Lipolysis in adipose tissue occurring after calving for lactation energy demands mobilizes fatty acid reserves together with the synthesis and generation of proteins (adipokines), lipid products, and free radicals, including ROS, and leads to an inflammatory response. In healthy cows with an adequate lipolytic response, antioxidant defenses contrast this inflammation threat through transcription and translation of complement and acute-phase proteins and enriching antioxidant defenses, such as glutathione peroxidase [140]. In cows with negative energetic balance (NEB), persistent ROS production depletes antioxidant systems, and OS develops causing inflammatory responses and impairing adipocyte response to insulin. This leads to a vicious circle where OS exacerbates lipolysis and inflammation, which further promotes OS [140,141]. The dynamics of these events evolve rapidly also because there is emerging evidence that immunity and cell metabolism are highly integrated, and metabolic stress impairs immunity [142,143]. Metabolic problems detected in the postpartum could also depend on inadequate management of the dry period. To evaluate this possibility, Tanigushi and colleagues [144] evaluated some blood parameters of cows in the dry period and associated with the onset of endometritis. Blood levels of glucose, BHB, calcium, and magnesium in dry periods were related to endometritis occurrence and, therefore, have been proposed as reliable indicators for predicting the risk of postpartum SCE. Additionally, a low IGF-1 serum concentration and a body condition score of less than 2.75 few weeks before calving were found to be associated with cytological endometritis [137].

### 6.3. Diagnosis of Endometritis and OS Markers

Focusing on the markers most closely related to OS, Table 1 provides a summary of the studies conducted on OS markers associated with endometritis in the cow and mare. Krishnan and colleagues [145] found higher plasma concentrations of NO and MDA in crossbred cows with SCE. In addition, cows with SCE had significantly higher H_2_O_2_ in isolated blood PMN as compared to healthy cows. Kaya and colleagues [146], scoring bovine endometritis in relation to clinical, ultrasound and cytological (cytobrush) evaluations, found that, in blood serum, NO and MDA levels progressively increased together with the severity of endometritis. Moreover, in cows with endometritis, TAC, assessed by ABST-based method, significantly decreased, whereas TOS did not show significant variations between healthy and sick cows. Siquera and colleagues [123] found that cows with endometritis showed an increase in serum levels of MDA, and hence, lipid peroxidation, as well as a higher MPO activity, used as an inflammatory marker and related to neutrophil activation. However, the activity of an antioxidant enzyme, such as catalase, was higher in cows with endometritis than in healthy cows. Among biomarkers evaluating the total buffering capacity, Emre and colleagues have recently applied the thiol/disulfide homeostasis (TDH) assay in cows with chronic endometritis [147]. Cows with endometritis showed a significant reduction in this indicator; this was attributable to a significant decrease in serum levels of native thiol and total thiol levels rather than to variations in serum disulfide levels.

Additionally, in mares, endometritis has been associated with oxidative processes. In particular, a significantly higher plasma concentration of MDA and lower glutathione peroxidase (GPx) activity in erythrocytes were found in Arabian mares with endometritis [153]. Other studies demonstrated, in mares with endometritis, an increase in MDA, NO metabolites, and estradiol, and a decrease in progesterone and TAC values [113]. In endometritis-susceptible mares, following insemination at the foaling heat, neutrophil activity increased together with MDA and fibrinogen plasma levels, whereas MPO was slightly lower in susceptible than in resistant mares [149]. Attempts to avoid physiological post-partum OS were carried out in Thoroughbred mares through dietary supplementation with antioxidants, such as α-tocopherol, during the peripartum period [154]. FRAP and ROMs levels were measured in blood samples of mares before, soon after, and one week after foaling. The serum α-tocopherol and FRAP levels did not differ between treated and control groups but were higher in mares > 10 years old. Mares receiving three natural insemination services showed lower values of α-tocopherol during postpartum compared to mares receiving only one service. However, serum ROMs did not differ between service classes.

OS biomarkers have been used to monitor the oxidative state of the uterine environment following treatments for endometritis. At the moment, one of the most discussed therapeutic aids in veterinary medicine is ozone (O_3_) therapy [155]. Although this treatment carries out a strong oxidizing activity, a small, well-calibrated, O_3_ dose may trigger several useful biochemical mechanisms and reactivate the antioxidant system, which in turn generates a therapeutic effect [156]. In particular, O_3_ therapy plays antimicrobial and anti-inflammatory effects, activates immune cells, and shows the ability to react with many compounds, such as phospholipids, lipoproteins, and microbial components. In healthy mares, three consecutive infusions of O_3_ in both solution and gaseous forms affected systemic OS markers, leading to a reduction in uric acid levels and TAC, together with an increase in TOS on days 3 and 6 following therapy. However, no changes were observed on albumin and LPO levels [148]. Mares with endometritis treated with O_3_ showed a tendency to increase the percentage of healing in microbiological analysis with the use of O_3_ in both oily or gaseous vehicles [157]. A distinctly different therapeutic approach but still able to reduce the oxidative events involved in the pathogenesis of endometritis was played by the platelet-rich plasma (PRP) treatment. Uterine infusion of PRP in jennies with endometritis induced a high *NF-kB* expression in mononuclear cells as well as downregulated *TRAF-1* and *MUC-1* gene expression in extracts of uterine fluid. Moreover, significant changes in OS biomarkers in both serum and uterine fluid were recorded after PRP treatment [152]. In particular, MDA and estradiol levels decreased, whereas TAC and progesterone levels increased progressively to 21 d post-treatment. A further therapeutic approach evaluated the effect of natural extracts, such as *Aegle marmelos* and *Murraya koenigii*, on endometritis in dairy cows [150]. Both treatments significantly decreased the bacterial load and PMN cell count, and affected the oxidative parameters by decreasing MDA and increasing ascorbic acid, reduced glutathione (R-GSH), and TAC; SOD only showed a tendential increase. Experimentally induced endometritis in animal models allowed to conduct of analytical investigations on organs and tissues under defined conditions as well as to verify the effect of treatments. In rats with induced endometritis, the activity of natural products, such as *Eucalyptus robusta* leaves crude extract, can positively influence the redox balance and comparative modulatory efficacy in many organs and tissues more effective than antibiotic treatments with cephalosporins (cefixime). In particular, animals treated with this natural extract showed lower levels of R-GSH in liver and brain, whereas SOD and catalase activities were significantly higher in some organs/tissues than in the antibiotic group [158].

## 7. Conclusions

In this study, we have provided an accurate description on (i) the role of the oxidative state and its alterations in animal organisms, (ii) the methods with which oxidative stress is usually evaluated, and (iii) the main molecular biomarkers and by-products associated with inflammation and oxidative stress. The etiopathogenesis of endometritis in cows and mares has been described considering its implications for the immune system. Attempts have, therefore, been described to tackle the diagnostics of this pathology with molecular approaches, which consider proteomics, transcriptomics, and metabolomics. Finally, attention was focused on the possible use of biomarkers of inflammation and oxidative stress measured in either the peripheral blood or the uterine fluid. Despite the limitations of blood tests due to their poor diagnostic specificity for the impossibility of attributing the origin of inflammatory products to the uterine site, these tests are increasingly used to check the effectiveness of endometritis treatments as well as to deepen scientific knowledge on the implications related to this pathology.

## Figures and Tables

**Table 1:** Main oxidative stress biomarkers associated with endometritis in cows and mares and grouped according to the detecting method.

	Abbreviation	Species	Based Method	References
**Total oxidants or total oxidant-derived substances**				
Total oxidant status	TOS	Bovine Equine	*o*-dianisidine *o*-dianisidine	[146] [148]
Nitric oxide metabolites	NOx (NO_2_ + NO_3_)	Bovine Equine	Griess react. (NO_2_ + NO_3_) Griess react. (NO_2_)	[145,146] [113]
Mieloperoxidase	MPO	Bovine Equine	MPO activity MPO release	[123] [149]
**Total buffering capacity or single-antioxidant substances**				
Total antioxidant capacity	TAC	Bovine Equine	ABTS DHBS DHBS ABTS	[146] [150,151] [113,152] [148]
Thiol/disulfide homeostasis	TDH	Bovine	NaBH_4_/DTNB	[147]
Catalase Glutathione peroxidase	CAT GPx	Bovine Equine	H_2_O_2_ decomposition cumene hydroperoxide	[123] [153]
Superoxide dismutase	SOD	Bovine	-	[150]
Reduced glutathione	R-GSH	Bovine	-	[150]
Albumin	Alb	Equine	bromocresol green	[148]
Uric acid	UA	Equine	enzymatic-Trinder	[148]
Fibrinogen	Fgn	Bovine Equine	heat precipitation heat precipitation	[123] [149]
Ascorbic acid	AA	Bovine	orthophosphoric acid	[150]
**Oxidative lipid damage**				
Malondialdeyde	MDA	Bovine Equine	TBARS TBARS	[123,145,146,150,151] [113,148,149,152,153]
